# Synthesis of thiolated chitosan and preparation nanoparticles with sodium alginate for ocular drug delivery

**Published:** 2012-07-18

**Authors:** Xuan Zhu, Meiqin Su, Shaoheng Tang, Lingsong Wang, Xinfang Liang, Feihong Meng, Ying Hong, Zhiran Xu

**Affiliations:** 1 School of Pharmaceutical Science, Xiamen University, Xiamen, China; 2 College of Chemistry & Chemical Engineering, Xiamen University, Xiamen, China

## Abstract

**Purpose:**

The goal of the present study was to synthesize mucoadhesive polymer – thiolated chitosan (TCS) from chitosan (CS), then prepared CS/TCS-sodium alginate nanoparticles (CS/TCS-SA NPs), determined which was more potential for ocular drug delivery.

**Methods:**

A new method for preparing TCS was developed, and the characteristics were determined using Fourier transform infrared spectroscopy and the degree of thiol immobilized was measured by Ellman’s reagent. Human corneal epithelium (HCE) cells were incubated with different concentrations of TCS for 48 h to determine the cell viabilities. CS/TCS-SA NPs were prepared and optimized by a modified ionic gelation method. The particle sizes, zeta potentials, Scanning electron microscopy images, mucoadhesion, in vitro cell uptake and in vivo studies of the two types of NP were compared.

**Results:**

The new method enabled a high degree of thiol substitution of TCS, up to 1,411.01±4.02 μmol/g. In vitro cytocompatibility results suggest that TCS is nontoxic. Compared to CS-SA NPs, TCS-SA NPs were more stable, with higher mucoadhesive properties and could deliver greater amounts of drugs into HCE cells in vitro and cornea in vivo.

**Conclusions:**

TCS-SA NPs have better delivery capability, suggesting they have good potential for ocular drug delivery applications.

## Introduction

Several challenges exist in the treatment of ocular diseases because eyes have special and complex anatomic structures. The main problem is to maintain an effective drug concentration at the site of action for an appropriate period of time, to improve the ocular bioavailability of a drug [1]. For this reason, much effort has been put into developing nanotechnology-based drug delivery systems to improve ocular availability of drugs by increasing the pre-corneal residence time [2-5]. Nanoparticles are solid colloidal particles with diameters ranging from 1 to 1,000 nm. Nanoparticles have excellent tissue penetration and persistence and for drug delivery, more targeted dosing and longer intervals between doses are beneficial to patients [6,7].

Chitosan (CS), a cationic polysaccharide, has been widely used in ophthalmic therapy [1]. CS is a mucoadhesive polymer that is a promising candidate for ocular therapy because of its biocompatibility, biodegradability, and low toxicity. Furthermore, CS has penetration-enhancing properties and it has attracted significant attention because it is a potential absorption enhancer for transport across the mucosal epithelia. However, CS has poor water solubility at pH >6 and this limits its effectiveness at absorption sites. Above pH 6, CS loses its positive-charge density, induces the formation of aggregates and precipitates [8].

To solve this problem, thiolated chitosan (TCS), comprising a new generation of mucoadhesive polymers – thiolated polymers or thiolmers [9] – has been produced. TCS provides much better mucoadhesive properties than CS, which can be explained by the formation of covalent disulfide bonds between the thiol groups and the mucus glycoproteins, which are much stronger than non-covalent bonds. Compared with CS, TCS has several advantageous features, such as significantly improved permeation and mucoadhesive properties. Moreover, soluble TCS displays gelling properties in situ at physiologic pH [10]. In this study, we chose thioglycolic acid as the thiolated reagent for TCS preparation.

Methods for TCS preparation are well known [11-15]. It is believed that larger amounts of immobilized thiol groups improve mucoadhesive properties [9] so we explored a new method to synthesize TCS, aimed at obtaining more effective and greater thiol group immobilization.

Sodium alginate (SA) is another interesting polysaccharide that has been used in several ocular delivery systems, either alone or in combination with other materials [16,17]. The formation of CS/SA nanoparticles, by the ionic gelation technique, has already been reported [18,19]. Nanoparticles have shown an effective encapsulating capacity and have been designed for prolonged delivery of drugs to the ocular mucosa [20].

In the present study, we first produced TCS using a new method and measured its characteristic by determining the degree of thiol group substitution using Ellman’s reagent and Fourier transform-infrared (FT-IR) analysis. We then prepared CS/TCS-SA nanoparticles (CS/TCS-SA NPs), optimized the formulation and analyzed nanoparticle characteristics, including particle size, zeta potential, scanning electron microscopy (SEM), and performed mucoadhesion studies with mucin. Furthermore, to confirm that TCS-SA NPs can deliver greater levels of drug into target sites than CS-SA NPs, we chose human corneal epithelium (HCE) cells to measure the degree of in vitro cell uptake, also, in vivo studies were performed in rat corneas by stereomicroscope and confocal laser scanning microscopy (CLSM).

## Methods

### Materials

Chitosan (low molecular weight, 20–300 cp (centipoise; 1% in 1% acetic acid), and degree of deacetylation 75%–85%), TGA (thioglycolic acid, ≥98%), Ellman's reagent (5,5′-dithiobis(nitrobenzoic acid), ≥98%), and mucin (from porcine stomach, Type III, bound sialic acid 0.5%–1.5%, partially purified powder) were obtained from the Sigma-Aldrich, St Louis, MO. EDAC·HCl (N-(3-dimethylaminopropyl)-N'-ethylcarbodiimide hydrochloride, 99%) and NHS (N-hydroxysuccinimide, 98%) were purchased from J&K China Chemical (Shanghai, China). FITC (fluorescein isothiocyanate, isomer 1, 95%) was provided by Alfa Aesar Chemical (Ward Hill, MA). DMF (N, N-dimethylformamide) and SA (sodium alginate of low viscosity (0.02 Pa·s) for a 1% solution at 20 °C) were purchased from Shanghai Chemical Co. Ltd (Shanghai, China). All other chemicals were reagent grade and were used as received.

### Animals and treatment

Male Wistar rats (220±30 g, purchased from the Shanghai SLAC Laboratory Animal Co. Ltd., Shanghai, China) were used for this study. The rats were kept in standard environment throughout the study as follows: room temperature 25±2 °C, relative humidity 60±10%, and alternating 12 h light-dark cycles (8 AM to 8 PM). All procedures were performed in accordance with the ARVO Statement for the Use of Animals in Ophthalmic and Cision Research.

### Synthesis of TCS

**Figure 1:**
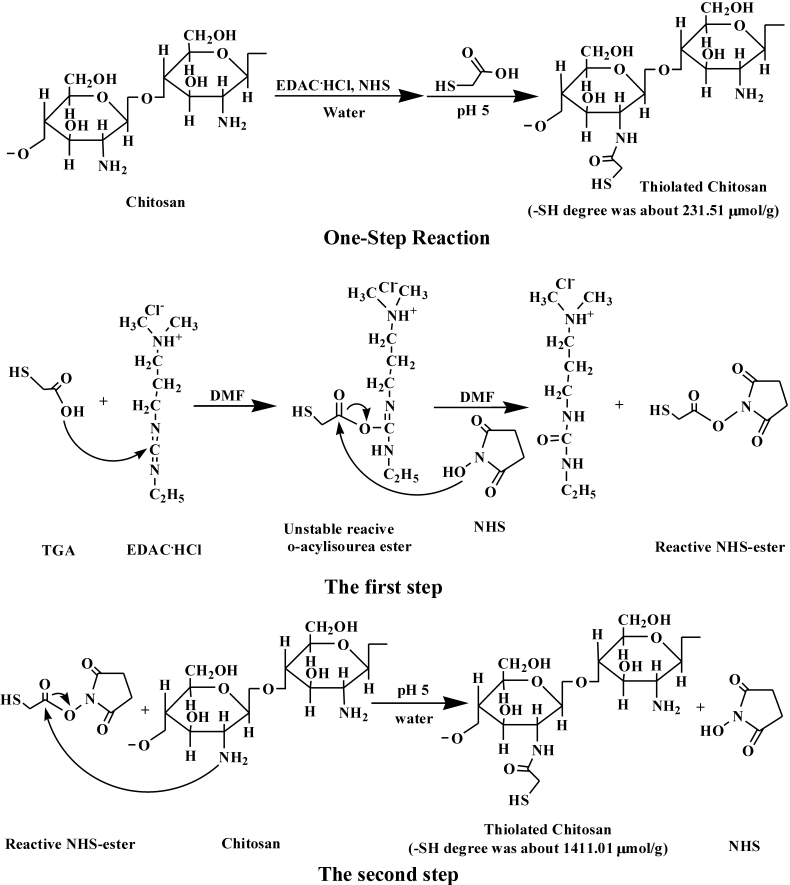
The reaction scheme of TCS synthesis, One-Step Reaction and The first step and The second step of the new method. Covalent attachment was achieved by the formation of amide bonds between the primary amino groups of CS and the carboxylic acid groups of TGA mediated by a carbodiimide (EDAC) and NHS. One-Step Reaction used water as reaction medium, while The first step of the new method used DMF as reaction medium to avoid the unstable O-acylisourea ester hydrolyzing in water, and obtained much higher thiol groups substituted in TCS, at 1411.01±4.02 versus 231.51±15.52 μmol/g.

Our new method includes two steps. The reaction scheme is shown in Figure 1. Briefly, in the first step, 1 mL of TGA, 3,500 mg of EDAC·HCl and 2,000 mg of NHS were added to a flask containing 2 ml DMF under constant stirring, overnight. After completion of the reaction, a reactive NHS-ester was produced. In the second step, 500 mg of chitosan was hydrated in 4 ml of 1 M HCl and dissolved by the addition of demineralized water to obtain a 2.5% solution of chitosan hydrochloride. Thereafter, the reactive NHS-ester was added dropwise into the chitosan hydrochloride solution, with the pH maintained at 5 using 10 M NaOH. The mixture was incubated at room temperature under continuous stirring overnight. To isolate the TCS, the polymer solutions were exhaustively dialysed in tubing (molecular weight cut-off 12 kDa; dialysis tubings, cellulose membrane; Shanghai Greenbird Co. Ltd [Shanghai, China]), first against 5 mM HCl, three times against 5 mM HCl containing 1% NaCl, and three times against 1 mM HCl at 8 °C in the dark. The controls were synthesized in the same way but omitting EDAC·HCl and NHS. The frozen aqueous polymer solutions of samples and controls were lyophilized (FreeZone, Labconco, Kansas City, MO), then stored at 4 °C for further use.

### Determination of the thiol groups in TCS by Ellman’s method

The degree of thiol group substitution in the modified polymers was determined spectrophotometrically using Ellman’s reagent, as described in a previous study [21]. Ellman’s reagent or 5, 5′-dithiobis (2-nitrobenzoate) (DTNB) is a symmetric aryl disulfide, which is very sensitive to the reaction of the thiol-disulfide interchange with a free thiol. Initially, 5 mg of each of the conjugates and controls dissolved in ultrapure water (PURELAB Classic UVF, ELGA LabWater, High Wycombe, Buckinghamshire, UK) to prepare 2 mg/ml solution. Then 250 μl aliquots were added to 250 μl of 0.5 M phosphate buffer (pH 8.0) and to 500 μl of Ellman’s reagent (0.4 mg/ml of DTNB in 0.5 mol/l phosphate buffer, pH 8.0). The sample was incubated at room temperature and shielded from light for 3 h. The resulting solution was then centrifuged at 18,000× g for 10 min. After that, 200 μl of the supernatant were transferred to a microtitration plate and the absorbance was measured at a wavelength of 450 nm with a Multimode Reader (Infinite M200; Tecan Group Ltd., Männedorf, Switzerland). A blank control was created with non-modified chitosan. A TGA standard curve was established between 0.125 and 2 μmol/ml, precisely following the same protocol used for sample determination.

### Characterization of TCS

The FT-IR (Fourier transform-infrared) spectra of chitosan and TCS were determined on a Nicolet avatar 330 FT-IR (Thermo Electron Corporation, Madison, WI) using the KBr (potassium bromide) method. TCS was confirmed by the presence of the characteristic peaks of newly formed amide bonds and thiol groups.

### Preparation of CS/TCS-SA NPs

Nanoparticles of CS or TCS with SA were prepared by a modified ionic gelation method as reported by Calvo et al. [22]. Based on preliminary experiments, CS was dissolved in 1% acetic acid aqueous solutions at various CS concentrations: 0.5, 1.0, 1.5 mg/ml, and the pH was adjusted using 1 M NaOH to pH 5.5. In contrast, TCS was easily soluble in aqueous solutions below pH 5 and we therefore chose ultrapure water as the solvent for TCS. SA was then dissolved in ultrapure water at concentrations of 0.625 or 1.25 mg/ml, using 1 M HCl to adjust the pH to 5.3. After the above steps, all the solutions of the materials were purified through a 0.45 μm microporous membrane filter. NPs were obtained by addition of SA solution (2 ml, pH 5.3) to aqueous solutions of polymers CS or TCS (8 ml) under magnetic stirring at room temperature. Samples were then visually analyzed for three kinds of phenomena: clear solution, opalescent suspension, and aggregates. The opalescent suspension was the objective when preparing the mucoadhesive nanoparticulate system.

### Optimization of CS/TCS-SA NPs

Nanoparticles were formed by interaction between the negative groups of alginate and the positively charged amino groups of CS or TCS. High concentrations of CS/TCS (1.5 mg/ml) and SA (2.5, 1.25 mg/ml) resulted in the formation of a high concentration of fibrous aggregates. In subsequent formulations, by reducing the SA concentration to 0.625 mg/ml and changing the concentration of CS/TCS from 0.5 to 1.5 mg/ml, we found the aggregation was reduced or even eliminated, respectively. Also sizes were significantly decreased and TCS-SA NPs had a smaller size than CS-SA NPs at the same concentration. It was observed that the concentration of CS/TCS (0.5 mg/ml) and SA (0.625 mg/ml) resulted in the formation of particles without aggregation, even after the particles were left overnight with stirring. This formulation was selected for subsequent studies and characterization as well as for entrapment of the model drug.

To determine cellular uptake, we chose FITC (fluorescein isothiocyanate) as a model drug encapsulated into the nanoparticles. FITC has excitation and emission peak wavelengths of approximately 494 nm and 520 nm, respectively. It is prone to photobleaching, which is suitable for tracking nanoparticles. The packed dye acts as a probe for NPs and offers a sensitive method for determining their intracellular uptake and retention. For FITC-loaded nanoparticles, 2 mg of FITC was dissolved in 100 μl of DMF, then added into the CS/TCS solution under gentle stirring before SA solution was added dropwise. The resultant NPs were collected by centrifugation (18,000× g, 60 min, 4 °C, Microfuge 22R; Beckman Coulter, Brea, CA), then the pellet was redispersed in water and lyophilized. All the operations were shielded from light because FITC is sensitive to light.

### Particle size analysis and zeta potential measurement

The mean size, size distribution and zeta potential of the prepared blank and FITC-loaded CS/TCS-SA NPs were determined by dynamic light scattering using a Zeta Nanosizer (Zetasizer Nano ZS, Malvern, UK).

### Scanning electron microscopy (SEM) studies

The surface morphology of FITC-loaded nanoparticles was imaged on a LEO 1530 scanning electron microscope (LEO GmbH, Munich, Germany) with a field emission electron gun. Nanoparticle suspensions were fixed on an aluminum disk and dried at room temperature. The dried nanoparticles were coated with gold metal using a sputter coater.

### Encapsulation efficiency estimation

The encapsulation efficiency (EE) was determined after isolating the NPs from a nanoparticle suspension containing free FITC by centrifugation (14,000× g, 60 min, 4 °C, Microfuge 22R; Beckman Coulter). The amount of free FITC in the supernatant was measured by fluorometric analysis (F-7000; Hitachi, Tokyo, Japan) at an excitation wavelength, E_x_, of 494 nm and emitted wavelength, E_m_, of 520 nm. FITC is a pH-sensitive fluorescent label indicator [23] so the supernatant was diluted with PBS (0.2 mol/l, pH 7.4) to ensure the assay maintained a constant pH. The EE of FITC was calculated using the following equation:

$$\text{EE (\%) = (1-}\frac{\text{Free FITC}}{\text{Total FITC}}\text{) x 100\%}$$

### Mucoadhesion studies with mucin

Equal volumes of mucin solution (0.4 mg/ml) and FITC-loaded CS/TCS-SA NPs were vortexed for 1 min and the zeta potential of the mixtures were measured by the Zeta Nanosizer.

### Cell culture

HCE cell line was cultured on a 100 mm cell culture plate in DMEM medium supplemented with 10% calf serum, 100 U ml^−1^ penicillin and 100 μg/ml streptomycin in 37 °C, 5% CO_2_.

### In vitro cytocompatibility studies

TCS was dispersed in a sufficient quantity of culture medium to prepare a series of concentrations from 100 to 1,000 ppm, with normal cell culture wells without polymers used as negative controls. For the in vitro cell toxicity assay, cells were plated in a 96-well plate at a density of about 1×10^4^ cells per well for 24 h. The medium was replaced by 0.2 ml of cell medium containing desired concentrations of TCS. After incubation for 12 h, cell viabilities were measured by the standard MTT (methyl thiazolyl tetrazolium) assay.

### In vitro cell uptake studies

Isolated FITC-loaded CS/TCS-SA NPs were resuspended in culture medium for fluorescence microscopy studies. HCE cells were plated on a 24-well plate at a density of ~0.5×10^4^ cells per well. After incubation in fresh medium for 24 h, cells were incubated with 0.5 ml of medium containing 100 ppm FITC of CS/TCS-SA NPs suspensions for 8 h. The cell medium was removed and the cells were washed before PBS buffer solution was added. The samples were then analyzed by fluorescence microscope (Eclipse Ti-U; Nikon, Tokyo, Japan).

### Detection of FITC loaded by CS/TCS-SA NPs in rat corneas

FITC-loaded CS/TCS-SA NPs were concentrated by centrifugation, and resuspended in a small volume of PBS to obtain 1 mg FITC per mL of the nanoparticle suspension. The naked FITC was dissolved in 10 μl DMF, then diluted with 5 ml PBS (pH 7.4) to gain the same concentration of 1 mg/ml with FITC-loaded CS/TCS-SA NPs suspension. We can ignore the effect of DMF to eyes for its trace amount. Normal conscious rats were randomly divided into three groups (n=5 per group), placed in a restraint box and 4 instillations of 15 μl of the FITC solution, FITC-loaded CS/TCS-SA NPs were topically administrated in the cul-de-sac of the right eye at 20 min intervals, while PBS was instilled in the left eye as control.

One and a half hour after the last instillation, the eyes were washed with PBS and examined in a stereomicroscope connected to a digital camera (Multizoom AZ100; Nikon, Tokyo, Japan). After that, rats were sacrificed and the corneas were excised, embedd in OCT (Sakura Finetek, Inc., Torrance, CA) medium and freezed in liquid nitrogen, then were performed on cryosections (6 μm thick) of the corneas. Latter, the samples were fixed in acetone at −20 °C for 10 min, blocked with DAPI (Vector, Burlingame, CA), mounted, and photographed using a confocal laser scanning microscope (Olympus Fluoview 1000; Olympus, Tokyo, Japan).

### Statistical analysis

All experiments were repeated at least three times. ANOVA (GraphPad Prism 5, GraphPad Software, Inc.; San Diego, CA) was used for statistical analysis. All the data are expressed as mean±SD and a p value <0.05 was deemed to be statistically significant.

## Results & Discussion

### Preparation of TCS

**Figure 2:**
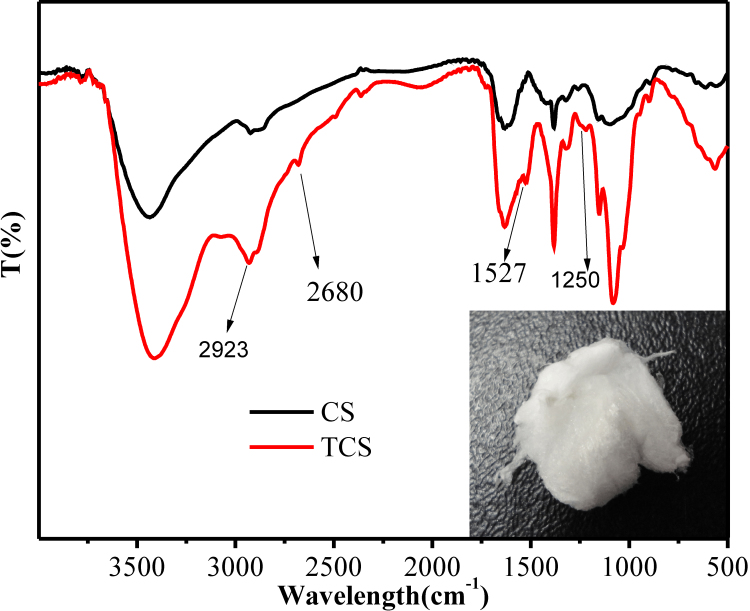
FT-IR spectra of CS/TCS and the appearance of TCS. The additional peaks of newly formed amide bond and peaks of thiol groups (from TGA) were observed: 1,250 cm^−1^ (S-C bond), 1,527 cm^−1^ (C=O double bonds of the amido bond), and 2,680 cm^−1^ (H-S bond). Both CS and TCS had absorption band at 2923 cm^−1^ peak corresponds to C-H bond. TCS appeared as a white, odorless fibrous structure after lyophilization.

It was demonstrated that TCS synthesized by the new method described above was more efficient than previous methods in which CS, TGA, coupling agent EDAC·HCl and NHS were mixed together in water. The scheme of the one-step reaction is shown in Figure 1. In contrast to the previously reported study, we chose DMF as the reaction medium rather than water. In addition, we split the single-step reaction into two steps. The first step produced the reactive NHS-ester [24], which was not only stable but effective for the subsequent reaction. The second step was the coupling reaction between the reactive NHS-ester and the primary amino groups of the cationic chitosan polymer. These modifications were designed to prevent the unstable O-acylisourea ester hydrolyzing in water and, moreover, the production of the reactive NHS-ester could increase the concentration of the target reactant over that in the one-step reaction. Compared with the one-step method, we obtained much higher thiol group immobilization in the polymer, at 231.51±15.52 versus 1,411.01±4.02 μmol/g. TCS appeared as a white, odorless fibrous structure after lyophilization, which was soluble in aqueous solution (shown in Figure 2).

### Characterization of TCS by FT-IR

The FT-IR spectra of CS and TCS are shown in Figure 2. Because the amino groups in chitosan reacted with the carboxyl groups of TGA, resulting in an amide bond, the additional peaks of this newly formed amide bond and peaks of thiol groups (from TGA) were observed. TCS has three characteristic peaks at 1,250, 1,527, and 2,680 cm^−1^, corresponding to the vibration of the S-C bond, C=O double bonds of the amido bond and the H-S bond, respectively, while the peak at 2,923 cm^−1^ is attributed to stretching of the C-H bond [14,25].

### In vitro cytocompatibility studies by MTT assay

**Figure 3:**
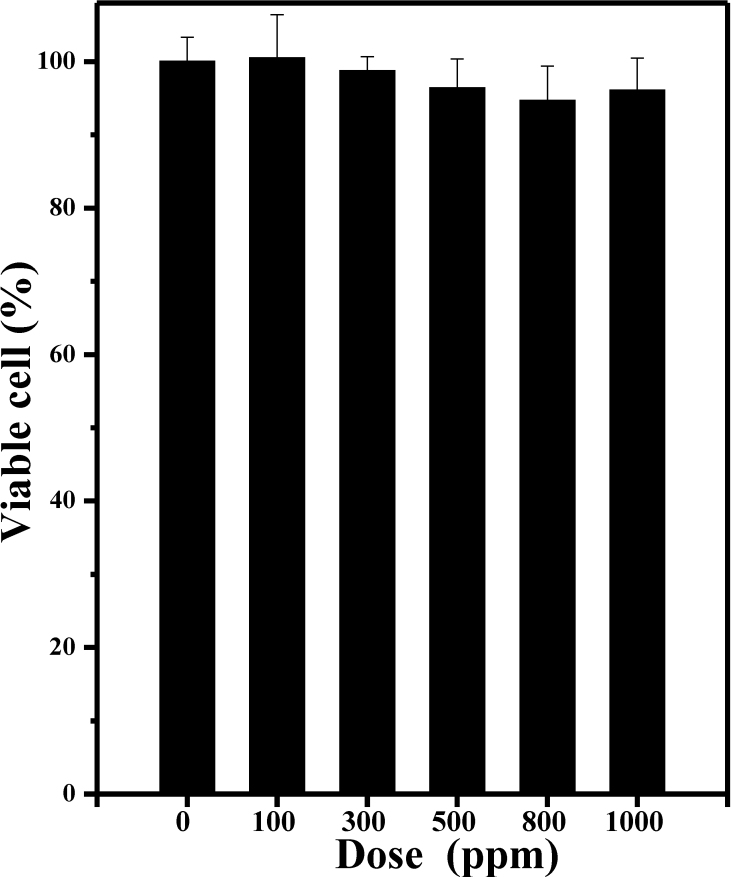
The relative cell viabilities of HCE cells incubated with different concentrations of TCS for 48 h (n=5). There was no significant difference in the percentage of cell viability in any of the concentrations of the TCS samples studied. About 94% cells were viable in all the different concentrations of TCS compared to the negative control. These results indicate that TCS has low toxicity toward HCE cells.

The relative cell viabilities of HCE cells incubated with different concentrations of TCS for 48 h were measured by the MTT assay. As shown in Figure 3 there was no significant difference in cell viability percentage for any of the TCS concentrations in the samples studied. It is evident from the figure that, compared with the negative control, almost 94% of the cells were viable at all the TCS concentrations. These results indicate that TCS has low toxicity toward HCE cells, an essential requirement for any material intended for use in biomedical applications.

### Physicochemical characterization of blank and FITC-loaded CS/TCS-SA NPs

The nanoparticles were formed as a result of the interaction of the positively charged amino groups of CS/TCS and negatively charged SA. For TCS, a portion of primary amine groups were coupled with thiol groups In other words, the resulting positively charged amino groups were decreased. This may explain how SA could interact with more TCS than CS without aggregation (Table 1). In addition, the TCS-SA NPs had a smaller size than CS-SA NPs at the same concentration, which was attributed to the formation of –S-S- bonds in or outside the polymers [10].

**Table 1:** Different concentrations of CS, TCS, and SA for NPs.

**Sample**	**CS^a^**	**TCS^a^**	**SA^a^**	**Aggregation^b^**	**Size^c^**	**PDI^d^**
1	1.5		2.5	++++++++++	-	-
2	1.5		1.25	++++++	-	-
3	1.5		0.625	+	617.5±54.9	0.52±0.11
4	1.0		0.625	+	513.9±12.1	0.53±0.07
5	0.5		0.625	No aggregation	338.1±15.9	0.25±0.01
6		1.5	2.5	++++++	-	-
7		1.5	1.25	+++	-	-
8		1.5	0.625	No aggregation	520.6±5.7	0.40±0.11
9		1.0	0.625	No aggregation	439.0±28.4	0.50±0.11
10		0.5	0.625	No aggregation	298.5±14.8	0.17±0.03

^a^Concentration in mg/ml. ^b^After overnight stirring. ^c^Size in nm and ^d^Polydispersity index were measured by dynamic light scattering using a Zeta Nanosizer. Experiments were performed in triplicate.

**Figure 4:**
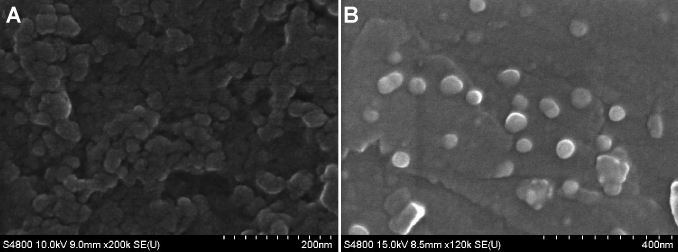
SEM microphotographs showed populations of spherical nanoparticles for both formulations (CS/TCS: 0.5 mg/ml, SA: 0.625 mg/mL). **A**: FITC-loaded CS-SA NPs; **B**: FITC-loaded TCS-SA NPs. Regular surfaces could be observed for both formulations and no important differences were denoted between the different formulations analyzed.

In the best possible formulation of the nanoparticles, CS/TCS (0.5 mg/ml) and SA (0.625 mg/ml) loaded FITC demonstrated a mean diameter of 471.0±6.4 and 265.7±7.4 nm for CS and TCS, respectively. The formulated particles were positively charged, which may be due to free residual amine groups not neutralized by the carboxyl groups of alginate (Table 2). For blank NPs, CS-SA NPs had a larger size but a lower zeta potential than TCS-SA NPs. This may have been caused by the formation of disulfide bonds in or outside the polymers at random [26], so that TCS-SA NPs become much tighter. Furthermore, disulfide bonds may change the conformation of TCS and block the interaction between amino groups and SA, leading to a higher zeta potential for TCS-SA NPs. In addition, as shown in Table 2, we observed that FITC-loaded CS-SA NPs were smaller than blank NPs, while FITC-loaded TCS-SA NPs were larger. It is also shown in the zeta potential column of the table, that both of the FITC-loaded NPs were smaller than the blank NPs. These differences may be due to the negative charges of FITC, so that in addition to the charge-charge interactions between CS/TCS and SA, FITC can also participate in the reaction. The more positively charged the amino groups, the more intensive was the interaction with FITC; therefore, the size was smaller and the zeta potential was lower. The higher zeta potential of the TCS-SA NPs indicated higher electrokinetic stability, as nanoparticles with a zeta potential of > 30 mV are more stable due to strong repulsive forces between particles that prevents aggregation. On the other hand, for the negatively charged cornea, higher positive charges are more effective. As shown in Figure 4, it was demonstrated that both formulations had spherically shaped particles and a high capacity for FTIC, which was helpful for the subsequent experiments.

**Table 2:** Physicochemical properties of blank or FITC-loaded NPs.

**Sample**	**CS^a^**	**TCS^a^**	**SA^a^**	**FITC^b^**	**Size^c^**	**PDI^d^**	**Zeta potential^e^**	**EE^f^**
5	0.5	-	0.625	-	338.1±15.9	0.25±0.01	+33.8±7.9	-
11	0.5	-	0.625	2	265.7±7.4	0.36±0.09	+29.5±4.1	84.2±1.8
10	-	0.5	0.625	-	298.5±14.8	0.17±0.03	+53.1±3.5	-
12	-	0.5	0.625	2	408.0±6.4	0.34±0.07	+49.2±2.3	92.1±1.9

^a^Concentration in mg/ml. ^b^FITC weighed in mg. ^c^Size in nm, ^d^Polydispersity index and ^e^Zetapotential in mV were measured by dynamic light scattering using a Zeta Nanosizer. ^f^Encapsulation efficiency of NPs measured by fluorometric analysis. Experiments were performed in triplicates.

### Mucoadhesion studies with mucin

**Figure 5:**
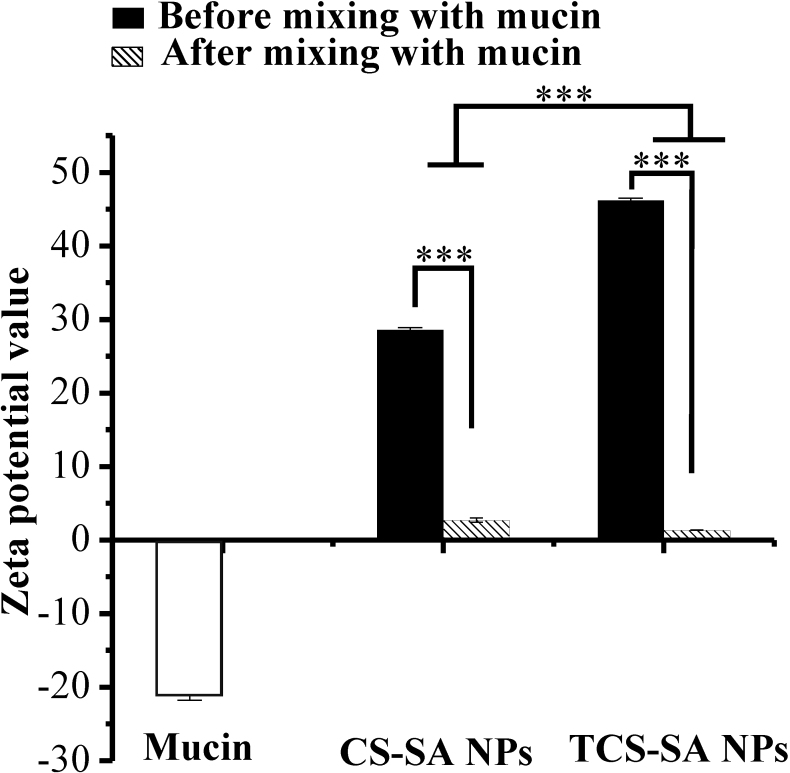
Zeta potential values obtained for mucin and FITC-loaded CS/TCS-SA NPs before and after incubation with mucin. The influence of mucin on the zeta potential of NPs revealed a reduction, and The gap in TCS-SA NPs was larger than in CS-SA NPs. Experiments were performed in triplicates (***p<0.0001).

Positively charged colloidal particles could increase electrostatic interaction between the particles and the negatively charged mucosal surface, thus leading to improved bioavailability and reduced side-effects. Addition of mucin reduced the zeta potential of the prepared NPs (Figure 5). This reduction could be due to the ionic interaction between negatively charged sialic acid residues in mucin and positively charged amino groups in CS/TCS. The intensity of the interaction also affected the mucoadhesive properties. As shown in Figure 5, the gap in TCS-SA NPs was larger than in CS-SA NPs, the disulfide bonds in the TCS-SA NPs may play an important role [10,26], which giving better mucoadhesive properties than those of CS-SA NPs. This property makes TCS-SA NPs a more versatile delivery system, which fulfills the requirements for applications in the ophthalmic field.

### In vitro cell uptake studies using fluorescent imaging

**Figure 6:**
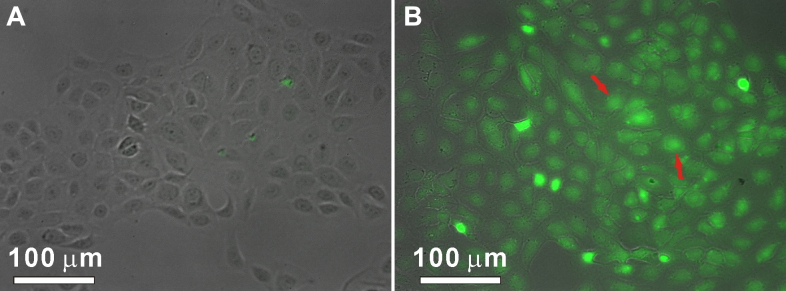
Fluorescent images of HCE cells incubated with same amount of FITC that loaded by CS/TCS-SA NPs for 8 h. The appearance of fluorescence signal of FITC (color of green) inside cells indicated the actual release of FITC from NPs. It showed that the in vitro cell uptake degree of NPs and TCS-SA NPs delivered much more FITC into cells than CS-SA NPs (as the arrows showed). **A**: CS-SA NPs; **B**: TCS-SA NPs (Scale bar: 100 μM).

In contrast to FITC loaded in NPs, free FITC molecules were highly fluorescent. We could take advantage of this property to monitor drug release through fluorescence signals. The release of FITC loaded in CS/TCS-SA NPs was studied in HCE cells. To observe the intracellular release of the drug in living cells by fluorescent microscopy, the cells were seeded on glass coverslips and incubated for 24 h. The cells were then further incubated in a medium containing FITC-loaded CS/TCS-SA NPs for 8 h. As revealed by fluorescence micrographs (Figure 6), intense fluorescence signals were observed inside the cells. Because the fluorescence of FITC was significantly quenched when loaded in NPs, the appearance of fluorescence signal inside cells indicated the actual release of FITC from NPs. As shown in Figure 6, it was demonstrated that the intracellular uptake of TCS-SA NPs was significantly greater than that of CS-SA NPs. This may be further confirmation that TCS-SA NPs are more adhesive and thus more effective as a potential system for ocular drug delivery, compared with CS-SA NPs.

### Detection of FITC loaded by CS/TCS-SA NPs in rat corneas

**Figure 7:**
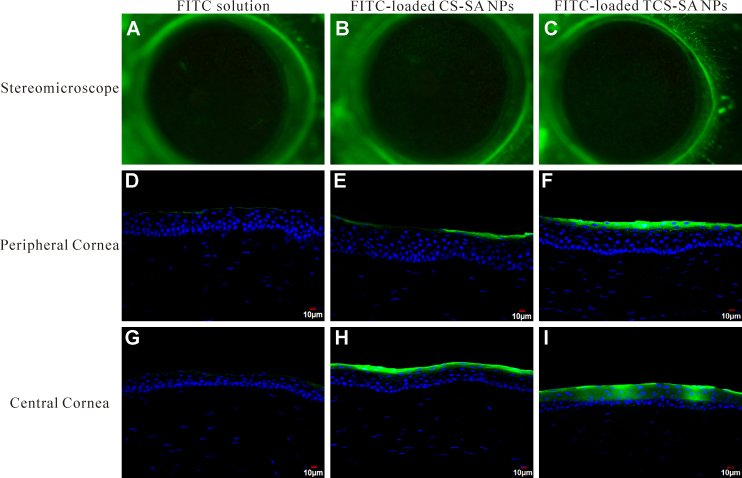
Detection of FITC loaded by CS/TCS-SA NPs in rat corneas. The rats were divided into 3 groups; FITC only, FITC-loaded CS-SA NPs, and FITC-loaded TCS-SA NPs. FITC were topically treated for 1.5 h. **A**-**C**: Representative images of stereomicroscope. **D**-**F**: Representative images of CLSM of peripheral cornea. **G**-**I**: Representative images of CLSM of central cornea. FITC displayed green fluorescence and nuclei were counterstained with DAPI (blue). Representative images from five individual animals are shown.

As shown in Figure 7, TCS-SA NPs can delivery more FITC into cornea than CS-SA NPs or FITC solution after the same operation. From the stereomicroscope, we detected that all three kinds of FITC can get into cornea, but the differences were not obviously. Whereas, from the concocal laser scanning microscope, nanoparticles can distinctly deliver more FITC into cornea than FITC solution, what’s more, TCS-SA NPs exhibited much better delivery ability than CS-SA NPs. Also, as shown in Figure 7, almost all FITC molecules were remained in corneal epithelium, which maybe due to the time of the treatment was too short, and latter more studies will be carried on.

#### Conclusion

A new method for preparation of TCS was established, and it achieved a high degree of thiol substitution, of up to 1,411.01±4.02 μmol/g, as determined by Ellman’s reagent. In addition, results of the MTT assay (almost 94% cell viability) indicated the safety of TCS toward HCE cells. The prepared CS/TCS-SA NPs were characterized by particle size, zeta potential, SEM and mucoadhesion studies. Overall, the results, including smaller size (265.7±7.4 to 471.0±6.4 nm), higher positive charges (49.2±2.3 to 29.5±4.1 mV) and higher mucoadhesion properties, suggest that TCS-SA NPs are more stable and more effective than CS-SA NPs as an ocular drug delivery system. The in vitro cell uptake study and in vivo experiments provide further evidence to support these conclusions.
